# eHealth Literacy, Awareness of Pandemic Infectious Diseases, and Healthy Lifestyle in Middle School Students

**DOI:** 10.3390/children8080699

**Published:** 2021-08-13

**Authors:** Sinyoung Choi, Kyung-Sook Bang, Da-Ae Shin

**Affiliations:** 1 College of Nursing, Seoul National University, Seoul 03080, Korea; csypass@snu.ac.kr (S.C.); olgar0108@snu.ac.kr (D.-A.S.); 2 College of Nursing, The Research Institute of Nursing Science, Seoul National University, Seoul 03080, Korea

**Keywords:** eHealth literacy, pandemic, infectious disease, COVID-19, healthy lifestyle, middle school students, adolescents

## Abstract

The coronavirus disease 2019 (COVID-19) pandemic is an unprecedented public health crisis worldwide. This pandemic has led to the spread of online misinformation, closure of schools, postponement of re-opening of schools, and restrictions on healthy outdoor activities. These, in turn, have caused a disruption in the daily life of middle school students. This study aimed to identify the relationships between eHealth literacy, awareness of pandemic infectious diseases, and healthy lifestyle in middle school students. For this purpose, we conducted a descriptive, correlational study in two cities in the capital of South Korea. A total of 138 middle school students completed self-reported questionnaires about eHealth literacy, awareness of pandemic infectious diseases, and healthy lifestyle. Middle school students displayed moderate levels of eHealth literacy (3.72 ± 0.97), awareness of pandemic infectious diseases (3.33 ± 0.92), and healthy lifestyle (2.98 ± 0.72). Positive correlations were found between eHealth literacy and awareness of pandemic infectious diseases (r = 0.44, *p* < 0.001), between eHealth literacy and healthy lifestyle (r = 0.52, *p* < 0.001), and between awareness of pandemic infectious diseases and healthy lifestyle (r = 0.38, *p* < 0.001). These findings suggest that eHealth literacy may be an important aspect of increasing the awareness about pandemic infectious diseases and forming healthy lifestyle habits in Korean middle school students during the COVID-19 pandemic.

## 1. Introduction

Since its first outbreak in late 2019, coronavirus disease 2019 (COVID-19) has spread rapidly despite various quarantine measures worldwide. COVID-19 was declared a pandemic, the highest level of alert for infectious diseases, leading to global disorder. The COVID-19 pandemic has also led to a so-called “infodemic” phenomenon, in which false health information about COVID-19 has spread rapidly through the Internet and the media. Therefore, the World Health Organization (WHO) warned that the infodemic may cause greater confusion than the COVID-19 pandemic [1], and the provision and use of accurate health information on the Internet has become an important global task [2].

As observed in the past cases of the H1N1 influenza and Middle East Respiratory Syndrome (MERS), the public’s desire for information increases rapidly when infectious diseases break out [3]. The public uses different mass media to satisfy their elevated demand, and the Internet is the main route to increasing access to various types of information online. The Internet helps the public to search and obtain information more easily [4]. According to the 2020 Survey on the Internet Usage in South Korea, 63.6% of health and medical information was obtained online after the COVID-19 outbreak, which represented the largest increase compared to before the COVID-19 outbreak (46.3%) among all internet search categories [5]. Furthermore, as information and messages delivered through the Internet directly and indirectly affect the thoughts and behaviors of the Internet users [4,6], the ability to use reliable and correct health information from the Internet is important for health-related behaviors and lifestyles during the pandemic [2,6].

eHealth literacy refers to the ability to seek, search, understand, and evaluate health-related information on the Internet and apply the knowledge to address health problems [7]. This ability plays an important role in the prevention and self-management of diseases and satisfaction of one’s need for health care [8,9]. Low eHealth literacy leads to difficulties in forming proper judgments of health-related knowledge acquired online, which subsequently causes health imbalances due to gaps and distortions in information [10], and undesirable health consequences [6]. eHealth literacy also plays a positive role in improving health knowledge and attitudes [8]. Adolescents who used mobile fitness applications showed increased exercise time and intention to maintain physical fitness compared to those who did not use the applications [11]. Additionally, US adults with high eHealth literacy maintained reasonable judgments about false superstitions, obtained accurate knowledge, and practiced infection prevention behaviors as compared to those with low eHealth literacy [6].

Adolescence is an important period of transition from childhood to adulthood with physical, emotional, cognitive, and socio-psychological maturation [12]. In particular, middle school students in early adolescence show the greatest growth and development. They are at the beginning of their cognitive development, and in forming and expressing their own thoughts and views on a variety of topics while reacting and adapting sensitively through interaction with their environment [13,14]. Thus, proper awareness and understanding of health issues and the development of health-promoting lifestyles during this period are essential to establish the basis for a healthy life [13,14,15]. Adolescents mainly use the Internet for leisure activities, communication, and education. In particular, use of the Internet for acquisition of data and information increased by 24.8% between 2015 and 2020 [16]. Moreover, the COVID-19 pandemic has led to increased online-based education and communication [5], and the use of and dependence on the Internet are expected to increase among middle school students.

Previous studies have reported that healthy lifestyles are determined by the evaluation of health information as well as individual attitudes, beliefs, and perceptions [14,17]. Another study found that adolescents’ knowledge of, attitudes to, and values about infectious diseases reflected awareness about the severity of infections and intention to engage in health protective behaviors [17]. To the best of our knowledge, however, no studies to date have examined the association between eHealth literacy, awareness of infectious diseases, and healthy lifestyle among this population.

Therefore, we assessed the relationships between eHealth literacy, awareness of pandemic infectious diseases, and healthy lifestyle among middle school students. In this study, we seek to provide basic data for strategies and measures on the development of desirable health habits among middle school students during the prolonged COVID-19 pandemic and to establish the basis to develop nursing interventions for pre-emptive measures and responses to pandemic infectious diseases in the future.

## 2. Materials and Methods

### 2.1. Research Design

This is a descriptive correlation study to assess eHealth literacy, awareness of pandemic infectious disease, and healthy lifestyles and their relationship among middle school students.

### 2.2. Subjects

Middle school students attending J middle school in Seoul and S middle school in Gyeonggi-do were selected through a convenience sampling method. The inclusion criteria were students who: (1) understood the purpose of the research and had no difficulty answering the questionnaires; (2) had agreed to participate and had obtained consent from their legal guardian. The exclusion criteria comprised unwillingness to participate in this study. The sample size required for two-tailed correlation analysis was calculated using the G*Power 3.1 program [18]. A total of 123 participants were required for a power of 0.80, effect size of 0.25, and significance of 0.05, as described by Kim and Suk [19]. The intent was to recruit a total of 144 participants, considering a dropout rate of 15%. One hundred forty-seven middle school students completed the questionnaire used in this study, and data from 138 students were included in the final analysis, excluding those of 9 students who had missing or incomplete values.

### 2.3. Research Tools

A structured questionnaire was used in this study. The questionnaire consisted of a total of 41 items including 6, 8, 9, and 18 items on general characteristics (school name, sex, grade, academic score, Internet usage time, and subjective health status), eHealth literacy, awareness of pandemic infectious diseases, and healthy lifestyles, respectively. For each tool, the research purpose was explained to the original authors to gain permission for the use of the tools.

#### 2.3.1. eHealth Literacy

eHealth literacy was assessed using a tool developed by Norman and Skinner [7] and modified and supplemented by Lee et al. [20] The tool consisted of eight items evaluated on a 5-point Likert scale from 1 point for “strongly disagree” to 5 points for “strongly agree.” A higher score indicated higher eHealth literacy. The Cronbach’s α was 0.88, 0.88 and 0.92 at the time of development, in the study by Lee et al. [20], and in this study, respectively.

#### 2.3.2. Awareness of Pandemic Infectious Diseases

Awareness of pandemic infectious diseases was assessed using a tool developed by Kim [21] and supplemented and modified by Park [22] based on the WHO’s Pandemic Influenza Risk Management WHO Interim Guidance. The tool was further modified and supplemented in this study in consideration of the COVID-19 pandemic. Specifically, “pandemic infectious disease” was changed to “COVID-19” in the tool, and the item “reasonability of classification and judgment of national infectious disease crisis level” was removed considering the level of middle school students who were the main participants in this study. The tool assessed individuals’ thoughts on pandemic infectious diseases. The items assessed whether the participant could provide a clear explanation of pandemic infectious diseases; the participant was interested in the process of pandemic infectious diseases; there was a possibility of pandemic infectious diseases; the response of the WHO and government was appropriate; the government had sufficient medication in stock; there would be a shortage of medicines in cases of outbreaks of pandemic infectious diseases; experts accurately predicted pandemic infectious diseases; and the severity of pandemic infectious diseases was high. The tool consisted of nine items in total, which were evaluated on a 5-point Likert scale from 1 point “strongly disagree” to 5 points “strongly agree,” and a higher score indicated that the participant agreed with the items related to pandemic infectious diseases. The Cronbach’s α of the tool was 0.59 in a study by Park [22] and 0.72 in this study.

#### 2.3.3. Healthy Lifestyles

Healthy lifestyles were assessed using a tool developed by Cho and Kim [23], which measured health behaviors and management of middle and high school students. The tool consisted of 18 items in total, broken down into five domains: mental health, exercise effort, nutrition and eating habits, hygiene management, and health responsibility in adolescents. The items were evaluated on a 4-point Likert scale (1 point: “strongly disagree”; 2 points: “disagree”; 3 points; “agree”; and 4 points: “strongly agree”). A higher score indicated a healthier lifestyle. At the time the tool was developed, internal composite reliability was greater than 0.7, and average variance extracted, which indicates the explanatory power through the size of variance for latent concepts, was greater than 0.5 in all domains, satisfying convergent and discriminant validities. Cronbach’s α of the tool was 0.82 in this study.

### 2.4. Data Collection and Ethical Considerations

This study was approved by the Institutional Review Board of Seoul National University (IRB No. 2102/001-009). To recruit participants, researchers explained the study’s purpose and method to the principals at two middle schools and asked for cooperation. After permission was obtained, announcements were posted in two classrooms for each grade in each school (12 classrooms in total). The informed consent form was written in a manner that the middle school students could fully understand. Written consent was obtained from all participants who voluntarily agreed to participate in the study and their legal guardians. Data were collected from 25 April to 24 May 2021, and the questionnaire was completed within approximately 30 min. At the time of data collection, the participants were told the purpose and methods of the study and informed that they could withdraw from the study at any time. Furthermore, the participants were told that the collected data would not be used other than for research purposes. The collected data were anonymized, and participants were told that data would be disposed of after three years and could only be accessed by the researchers.

### 2.5. Data Analysis

Data were analyzed using the SPSS/WIN 25.0 program. The normality of the distributions of variables was confirmed using the Kolmogorov–Smirnov test, and parametric analyses were used to explore the data. The general characteristics, eHealth literacy, awareness of pandemic infectious diseases, and healthy lifestyles were expressed using real number/percentage and mean/standard deviation. The independent *t*-test and one-way analysis of variance were conducted for assessment of differences in eHealth literacy, awareness of pandemic infectious diseases, and healthy lifestyles according to the general characteristics, followed by the Scheffé post hoc test. In addition, effect size values were calculated to confirm the practical significance. Pearson’s correlation coefficients were used to analyze correlations between eHealth literacy, awareness of pandemic infectious diseases, and healthy lifestyles.

## 3. Results

### 3.1. General Characteristics of the Subjects

A total of 94 female (68.1%) and 44 male (31.9%) middle school students were included in this study. Sixteen (11.6%), 53 (38.4%), and 69 (50.0%) participants were in first, second, and third year of middle school, respectively. Twenty-four (17.4%) students had low academic scores, while 88 (63.8%) and 26 (18.8%) students had middle and high academic scores, respectively. The greatest number of participants used the Internet for 1–3 h (44 participants, 31.9%), followed by 3–5 h (43 participants, 31.1%), and 5–7 h (29 participants, 21.0%). Twenty-two (15.9%), 40 (29.0%), and 58 (42.0%) participants had “very good,” “good,” and “moderate” subjective health status, respectively, with most participants having moderate or better subjective health status (Table 1).

### 3.2. eHealth Literacy, Awareness of Pandemic Infectious Diseases, and Healthy Lifestyles among Participants

The average score for eHealth literacy of middle school students was 29.75 ± 6.28 out of 40 points, while the average score for awareness of pandemic infectious diseases and for healthy lifestyles was 29.97 ± 4.62 out of 45 points and 53.56 ± 6.66 out of 72 points, respectively. The mean scores of the items, eHealth literacy, awareness of pandemic infectious diseases, and healthy lifestyles were 3.72 ± 0.97 out of 5 points, 3.33 ± 0.92 out of 5 points, and 2.98 ± 0.72 out of 4 points, respectively (Table 2).

For eHealth literacy, the item “I know how to find useful health resources on the Internet without difficulty” had the highest score at 4.03 ± 0.87 points, while the item “I can distinguish high-quality health resources on the Internet” had the lowest score at 3.32 ± 1.08 points. For awareness of pandemic infectious disease, the item “If a pandemic infectious disease occurs in the future, do you expect the severity of the disease to be very high considering the current COVID-19 situation?” had the highest score at 3.98 ± 0.91 points. In contrast, the item “do you think the government has secured enough medicine (vaccines and anti-viral drugs) for the pandemic?” had the lowest score at 2.75 ± 0.87 points.

For healthy lifestyle, the mean scores for mental health, exercise effort, nutrition and eating habits, hygiene management, and health responsibility were 3.29 ± 0.51, 2.67 ± 0.83, 2.32 ± 0.50, 3.54 ± 0.44, and 2.75 ± 0.57 points, respectively. The mean score for hygiene management was the highest, while the mean score for nutrition and eating habits was the lowest. In particular, the item “I shower and wash my body regularly” had the highest score at 3.69 ± 0.52 points. In contrast, the item “I do not eat processed and instant food products containing preservatives and artificial additives” showed the lowest score at 1.78 ± 0.73 points.

### 3.3. Comparisons of eHealth Literacy, Awareness of Pandemic Infectious Diseases, and Healthy Lifestyles According to General Characteristics

Differences in eHealth literacy, awareness of pandemic infectious diseases, and healthy lifestyles were assessed according to general characteristics (Table 1). eHealth literacy was significantly different according to grade (F = 6.04, *p* = 0.003) and academic score (F = 17.52, *p* < 0.001). Academic score had a large effect size of 21% (η^2^ = 0.21), and grade showed a medium effect size (η^2^ = 0.08) on eHealth literacy. eHealth literacy was higher among third-year students than among first-year students and among those with a greater academic score. Awareness of pandemic infectious diseases differed significantly according to the grade (F = 4.25, *p* = 0.016, η^2^ = 0.06) and was higher among third-year students than among first-year students. The eta-squared value for grade represented a medium effect size of 6%. Healthy lifestyles differed significantly according to academic score (F = 8.21, *p* < 0.001, η^2^ = 0.11), Internet usage time (F = 3.59, *p* = 0.008, η^2^ = 0.10), and subjective health status (F = 5.15, *p* = 0.001, η^2^ = 0.13). Academic score, Internet usage time and subjective health status showed a medium effect size on awareness of pandemic infectious diseases. Students with a higher academic score, less Internet usage time, and better subjective health status had a healthy lifestyle.

### 3.4. Correlations between eHealth Literacy, Awareness of Pandemic Infectious Diseases, and Healthy Lifestyle

eHealth literacy showed a positive correlation with awareness of pandemic infectious diseases (r = 0.44, *p* < 0.001) and healthy lifestyles (r = 0.52, *p* < 0.001). While awareness of pandemic infectious diseases was positively correlated with healthy lifestyles (r = 0.38, *p* < 0.001) (Table 3).

## 4. Discussion

The unprecedented global public health crisis caused by COVID-19 is accompanied by a large-scale infodemic and has led to closure of schools, postponement of re-opening of schools, beginning of the online classes, and restrictions on outdoor activities. This has caused confusion in the daily life of middle school students and is likely to have negative effects on their health management and desirable lifestyles. Thus, this study assessed eHealth literacy, awareness of pandemic infectious diseases, and healthy lifestyles among middle school students and their relationships in order to provide basic data for the healthy lifestyles of middle school students.

In our study, eHealth literacy of middle school students was 29.75 out of 40 points, which was similar to or higher than that of college students [24] and patients with chronic diseases [25]. This finding is consistent with previous studies that reported that eHealth literacy was higher among the younger age groups [10,20,26]. In particular, middle school students born in the late 2000s, who are the so-called “generation Z” and “digital natives,” are more familiar with the digital environment and access and use of information technology, which may have affected their eHealth literacy scores.

However, a detailed assessment showed that while the students had the highest “ability to search for useful health information on the Internet,” they showed the lowest “ability to evaluate health information obtained on the Internet.” This suggests that, although middle school students in South Korea are in an environment with easy access to online eHealth information due to the high penetration rate of the Internet, they lack the ability for critical assessment and application of the acquired health information. In fact, previous studies also showed that adolescents are able to access abundant health information on the Internet while lacking the capacity to find and distinguish reliable information [27,28], which supports our findings.

We observed that eHealth literacy was significantly different depending on the grade and academic score, which was consistent with the findings of Park [28]. However, contrary to a study of patients with chronic diseases by Kim et al. [25], there was no significant difference in eHealth literacy according to subjective health status and Internet usage time. This may be attributed to the relatively satisfactory health level and low interest in health of middle school students who show a passive attitude in searching for health information, compared to patients with chronic diseases who require continuous health management. This finding suggests that the eHealth literacy of middle school students can only be improved through a combination of quantitative expansion such as Internet usage time and penetration rate and qualitative expansion including the ability to think logically and judge information on the Internet. However, previous studies reported differences in eHealth literacy according to the presence of diseases [28] and showed that subjective health status affects the level of interest and use of health information, which subsequently affected eHealth literacy [25,29]. Therefore, further assessment on eHealth literacy according to health outcomes and interest in health in middle school students would be necessary.

In this study, middle school students scored 29.97 out of 45 points for awareness of pandemic infectious diseases, which was similar to the findings of a study that used the same tool [22]. Among the nine items, the score for awareness of “prediction of the likelihood and severity of pandemic infectious disease in the future” was the highest, while awareness of “appropriateness of medicine stock by the government” was the lowest. This may be attributed to the current unprecedented effects of COVID-19 on not only society and the economy worldwide but also the daily life of individuals with middle school students directly affected by contactless classes and closure of schools. In particular, given the experience of other pandemic infectious diseases such as H1N1 influenza and MERS, transparent disclosure of information on COVID-19 and active quarantine measurements could have affected middle school students’ attitudes toward and risk perception of pandemic infectious diseases. Additionally, the lack of a cure (due to the nature of a pandemic infectious disease), difficulties in developing a vaccine, and limited vaccination due to limited quantity may have had negative effects on the awareness.

We observed that awareness of pandemic infectious diseases differed between students in different grades. Those in third year had a higher awareness than those in first year. Most previous studies were conducted on adults and health care workers and were limited to knowledge, attitude, and ethical awareness on pandemic infectious diseases. Thus, direct comparison between our findings and those of previous studies is limited. Regardless, our results agreed with those of a study that reported significant differences in awareness of pandemic infectious diseases according to education level among healthcare providers [22] and another study that demonstrated that senior university students majoring in allied health had superior knowledge and attitude with regard to MERS than those in other years [30]. However, there was no difference in awareness of pandemic infectious disease according to academic score in our study. This indicates that the middle school students may lack the capacity to correctly identify and judge pandemic infectious diseases regardless of academic score.

Lastly, the healthy lifestyle score was 53.56 out of 72 points in our study. No previous study used the same measurement tool to evaluate middle school students, and it is thus difficult to directly compare our findings to those of other studies. However, Kim [19] used an adolescent health promoting lifestyle profile, which consisted of five domains: personal hygiene and health responsibility, nutrition and eating habits, exercise, stress management and interpersonal relationships, and self-realization, and reported a score of 53.1 points, which is similar to our finding. The students in our study had a mean score of 2.32 points (out of 4) for “nutrition and eating habits,” which was the lowest among all sub-domains. This was consistent with a previous study [19] in which “nutrition and eating habits” received the lowest score among other sub-domains of health promotion behavior for middle school students. Given that a high preference for instant and processed food products and inadequate intake of vegetables hinders the growth and development of adolescents, the role of health professionals in promoting healthy eating habits is fundamental.

On the other hand, “hygiene management” had a mean score of 3.54 points, the highest score of all five sub-domains. This was also higher than that observed in a previous study of the same participant group (i.e., middle school students) [19]. It is interpreted that emphasis on strict hygiene rules including washing hands, wearing a mask, and cough etiquette to prevent the spread of COVID-19 led to an increase in the practice of hygiene management among middle school students. The 2020 Youth Health Behavior Survey conducted by the Korea Disease Control and Prevention Agency also revealed that personal hygiene-related behaviors of adolescents increased by 12.0% between 2019 and 2020 [31], which agrees with our observation.

There were also significant correlations between eHealth literacy, awareness of pandemic infectious diseases, and healthy lifestyles among middle school students. This finding is consistent with studies that showed greater individual knowledge, attitudes, and protection behavior related to infectious diseases among those with higher eHealth literacy [6] and that reported that the ability to use online health information improves self-health management ability and is an important factor affecting exercise and eating habits [9, 32]. Moreover, this finding indicates that eHealth literacy may be an important factor affecting the awareness of pandemic infectious diseases and development of healthy lifestyles among middle school students who have many opportunities to acquire information from the Internet. Furthermore, awareness of pandemic infectious diseases was positively correlated with healthy lifestyles. This agrees with a study by Kim et al. [4] who reported that cognitive responses and attitudes related to pandemic infectious diseases are factors influencing preventive behavior intentions. Given that behavioral intentions are generally determined by subjective beliefs and awareness of individuals about behaviors, increased awareness, and interest in disease during the COVID-19 pandemic may have influenced the healthy lifestyles of middle school students.

The role of healthcare providers is critical to maintain and to promote children’s health, especially in this public health crisis. Our findings suggest that it is important to improve eHealth literacy and to raise awareness of new infectious diseases to promote healthy lifestyles of middle school students. In particular, it is necessary to develop and to implement systematic educational programs to improve their ability to judge and to critically accept high-quality and reliable information. In addition, strategies should be established to motivate middle school students to adhere to healthy lifestyles through active and regular measures that can maintain continuous interest in new infectious diseases.

Several limitations need to be considered in interpreting this study’s findings. First, this study was only conducted on students from two middle schools in South Korea, which provided close to the minimum sample size but was not randomly selected. Thus, the results cannot be generalized. Moreover, other exogenous variables, including parent-related variables and socioeconomic status that could affect the variables of interest, were not controlled. Therefore, further research into the relationships between eHealth literacy, awareness of pandemic infectious diseases, and healthy lifestyles is necessary while considering other environmental variables that may affect these factors.

## 5. Conclusions

This study assessed eHealth literacy, awareness of pandemic infectious diseases, and healthy lifestyles and their relationships among middle school students. The significant finding of this study is that awareness of pandemic infectious diseases may be an important factor affecting the practice of health behaviors in pandemic situations (i.e., the COVID-19 pandemic). Considering that future education, not to mention current education, will be gradually offered online and the nth wave of COVID-19 and emergence of new infectious diseases cannot be ruled out, more specific and organizational strategies and various nursing interventions for improved awareness of pandemic infectious disease and eHealth literacy would be necessary in the future to promote a healthy lifestyle among middle school students.

## Figures and Tables

**Table 1:** Comparisons of eHealth literacy, awareness of pandemic infectious diseases, and healthy lifestyles according to general characteristics. (*n* = 138).

Variables	Categories	*n* (%)	eHealth Literacy				Awareness of Pandemic Infectious Diseases				Healthy Lifestyles			
			M ± SD	t or F(*p*)	Scheffe’s Test	ES	M ± SD	t or F(*p*)	Scheffe’s Test	ES	M ± SD	t or F(*p*)	Scheffe’s Test	ES
Sex	Female	94 (68.1)	3.79 ± 0.77	1.78		0.33 *	3.37 ± 0.50	1.42		0.26 *	2.98 ± 0.39	0.43		0.08 *
	Male	44 (31.9)	3.55 ± 0.81	(0.077)			3.24 ± 0.54	(0.159)			2.96 ± 0.32	(0.671)		
Grade	1st ^a^	16 (11.6)	3.16 ± 0.66	6.04	a < c	0.08 ^†^	3.00 ± 0.48	4.25	a < c	0.06 ^†^	2.93 ± 0.33	0.45		0.01 ^†^
	2nd ^b^	53 (38.4)	3.66 ± 0.83	(0.003)			3.32 ± 0.48	(0.016)			2.95 ± 0.39	(0.639)		
	3rd ^c^	69 (50.0)	3.88 ± 0.72				3.41 ± 0.52				3.00 ± 0.36			
Academic	Low ^a^	24 (17.4)	3.26 ± 0.71	17.52	a < b < c	0.21 ^†^	3.19 ± 0.38	1.47		0.02 ^†^	2.88 ± 0.38	8.21	a < b < c	0.11 ^†^
score	Middle ^b^	88 (63.8)	3.64 ± 0.70	(<0.001)			3.34 ± 0.51	(0.235)			2.93 ± 0.36	(<0.001)		
	High ^c^	26 (18.8)	4.39 ± 0.71				3.43 ± 0.60				3.22 ± 0.27			
Internet	<1 ^a^	3 (2.2)	4.00 ± 0.88	0.57		0.02 ^†^	3.70 ± 1.16	1.34		0.04 ^†^	3.15 ± 0.74	3.59	b > e	0.10 ^†^
usage time	1~<3 ^b^	44 (31.9)	3.67 ± 0.89	(0.687)			3.44 ± 0.56	(0.258)			3.08 ± 0.37	(0.008)		
(hour)	3~<5 ^c^	43 (31.1)	3.68 ± 0.71				3.30 ± 0.45				2.97 ± 0.33			
	5~<7 ^d^	29 (21.0)	3.88 ± 0.69				3.23 ± 0.51				2.99 ± 0.34			
	≤7 ^e^	19 (13.8)	3.60 ± 0.85				3.24 ± 0.41				2.72 ± 0.33			
Subjective	Very bad ^a^	3 (2.2)	3.41 ± 1.05	1.49		0.04 ^†^	3.26 ± 0.32	0.19			2.67 ± 0.35	5.15	c < d < e	0.13 ^†^
health	Bad ^b^	15 (10.9)	3.99 ± 0.54	(0.209)			3.30 ± 0.50	(0.945)			2.86 ± 0.39	(0.001)		
status	Moderate ^c^	58 (42.0)	3.56 ± 0.86				3.32 ± 0.42				2.87 ± 0.34			
	Good ^d^	40 (29.0)	3.85 ± 0.77				3.38 ± 0.59				3.11 ± 0.34			
	Very good ^e^	22 (15.9)	3.76 ± 0.68				3.28 ± 0.64				3.14 ± 0.35			

Note: M = mean; SD = standard deviation; ES = effect size; * Cohen’s *d*; ^†^ Eta-squared (η^2^). ^a–e^ Superscript letters represent statistical differences between groups (*p* < 0.05).

**Table 2:** The degrees of eHealth literacy, awareness of pandemic infectious diseases, and healthy lifestyles of middle school students. *(n* = 138).

Variables	M ± SD	Min–Max	Convert M ± SD *	Reference Range
eHealth literacy (total = 40)	29.75 ± 6.28	8.0–40.0	3.72 ± 0.97	1–5
Awareness of pandemic infectious diseases (total = 45)	29.97 ± 4.62	18.0–36.0	3.33 ± 0.92	1–5
Healthy lifestyles (total = 72)	53.56 ± 6.66	21.1–72.0	2.98 ± 0.72	1–4

Note: * converting scale score.

**Table 3:** Correlations among eHealth literacy, awareness of pandemic infectious diseases, and healthy lifestyles. (*n* = 138).

Variables	eHealth Literacy	Awareness of Pandemic Infectious Diseases	Healthy Lifestyles
	r (*p*)		
eHealth literacy	1		
Awareness of pandemic infectious diseases	0.44 (<0.001)	1	
Healthy lifestyles	0.52 (<0.001)	0.38 (<0.001)	1

## Data Availability

Not applicable.
